# Honors to American Physicians

**Published:** 1880-02

**Authors:** 


*Honors to American Physicians.*—An interesting ceremony took place at St. John's Hospital, Beirut, Syria, on the 31st of last October, in the presentation of crosses of merit and knighthood in three German princely orders to Drs. Van Dyck, Wortabet, and Post, who constituted the medical staff of the hospital. These crosses came from the king of Prussia and the Dukes of Mecklenburg and Saxony. Dr. Van Dyck was decorated with the Prussian Order of the Crown, Dr. Wortabet with the golden cross of merit of the Order of the Wendish Crown, and Dr. Post with the Cross of the Knight of the Order of the Dukes of Saxony. Two of the knighted gentlemen are Americans—Drs. Van Dyck and Post. Dr. Geo. E. Post is a son of Dr. Alfred C. Post, of New York, and has already won a reputation throughout Syria and the Levant as an eminent surgeon. Dr. George E. Post is also a graduate of the Baltimore College of Dental Surgery of the Class of 1864.

